# Transcriptional dysregulation of TRIM29 promotes colorectal cancer carcinogenesis via pyruvate kinase-mediated glucose metabolism

**DOI:** 10.18632/aging.202414

**Published:** 2021-01-20

**Authors:** Jing Han, Zitong Zhao, Nan Zhang, Yang Yang, Liying Ma, Li Feng, Xue Zhang, Jing Zuo, Zhisong Fan, Yudong Wang, Yongmei Song, Guiying Wang

**Affiliations:** 1 Department of Medical Oncology, Hebei Medical University Fourth Affiliated Hospital and Hebei Provincial Tumor Hospital, Shijiazhuang 050000, Hebei, P.R. China; 2 State Key Laboratory of Molecular Oncology, National Cancer Center/National Clinical Research Center for Cancer/Cancer Hospital, Chinese Academy of Medical Sciences and Peking Union Medical College, Beijing 100021, P.R. China; 3 Department of Thoracic Surgery, Hebei Medical University Fourth Affiliated Hospital and Hebei Provincial Tumor Hospital, Shijiazhuang 050000, Hebei, P.R. China; 4 Department of General Surgery, Hebei Medical University Fourth Affiliated Hospital and Hebei Provincial Tumor Hospital, Shijiazhuang 050000, Hebei, P.R. China; 5 Department of General Surgery, The 3rd Affiliated Hospital of Hebei Medical University, Shijiazhuang 050000, Hebei, P.R. China

**Keywords:** colorectal cancer, TRIM29, transcriptional regulation, PKM1, glucose metabolism

## Abstract

Targeted molecular therapy is the most effective treatment for cancer. An effective therapeutic target for colorectal cancer (CRC) is urgently needed. However, the mechanisms of CRC remain poorly understood, which has hampered research and development of CRC-targeted therapy. TRIM29 is a ubiquitin E3 ligase that has been reported as an oncogene in several human tumors. In this study, we show that increased levels of TRIM29 were detected in CRC compared with normal tissues and were associated with poor clinical outcome, advanced stage and lymph node metastasis, particularly those with right-sided colorectal cancer (RSCC). Notably, GATA2 (GATA Binding Protein 2) transcriptionally repressed TRIM29 expression. The loss of GATA2 and high expression of TRIM29 occur more frequently in RSCC than in left-sided colorectal cancer (LSCC). Functional assays revealed that TRIM29 promotes the malignant CRC phenotype *in vitro* and *in vivo*. Mechanistic analyses indicate that TRIM29 promotes pyruvate kinase (mainly PKM1) degradation via the ubiquitin-proteasome pathway. TRIM29 directly targets PKM1 to reduce PKM1/PKM2 ratio, which results in PKM2-mediated aerobic glycolysis (Warburg effect) acting as the dominant energy source in CRC. Our findings suggest that TRIM29 acts as a tumor promoter in CRC, especially in RSCC, and is a potential therapeutic target for CRC treatment.

## INTRODUCTION

The incidence and mortality of colorectal cancer (CRC) ranks third and fourth among malignant tumors worldwide [1]. In China, the incidence and mortality of CRC have shown an increasing trend in recent years [2]. CRC has become a disease that seriously threatens human health. Surgery combined with chemotherapy and targeted therapy is the main treatment for CRC. To date, the driver gene and the mechanism for CRC are still not clear; hence, targeted molecular therapies for CRC are highly limited; the only available regimens involve targeting EGFR and antiangiogenesis drugs. The prognostic factors for CRC also need to be improved. Clinically, carcinoembryonic antigen (CEA) and the TNM staging system are now the main predictors for CRC [3, 4], but high CEA levels are not specific to CRC [5]. Therefore, it is urgent to seek effective biomarkers that can predict prognosis and act as targets for treating CRC.

Proteins are the main functional components of the body and are involved in almost every cellular process. A growing amount of evidence has demonstrated the practicality of proteins as biomarkers and targets for tumors [6]. It has been reported that HER2 overexpression is associated with poor outcome for CRC patients [7], and HER2 has become a potential target for CRC treatment. Recent studies revealed that malignant behavior is regulated by glycometabolism in various cancers, for example, pyruvate kinase M2 (PKM2) contributes to cell growth in gastric cancer via aerobic glycolysis. It is a new strategy for targeted molecular therapies to interfere with key proteins in glucose metabolism. TRIM29 is a ubiquitin E3 ligase that has been reported to induce EMT in CRC by activating the Wnt/β-catenin signaling pathway via upregulation of CD44 expression [8]. However, we do not know whether TRIM29 participates in glucose metabolism in CRC.

Our previous work has already identified differentially expressed genes (DEGs) (GEO accession number: GSE110204) between CRC tissues and matched adjacent normal tissues [9]. In this work, we found that of the DEGs, TRIM29 is more highly expressed in tumors than in adjacent normal tissues and is also associated with lymph node metastasis, advanced stage and poor prognosis of CRC. More interestingly, TRIM29 is much more highly expressed in right-sided colorectal cancer (RSCC) than in left-sided colorectal cancer (LSCC). TRIM29 is negatively transcriptionally regulated by GATA2. TRIM29 promotes the CRC malignant phenotype by regulating PKM1 associated glucose catabolism. Overall, our study reveals the critical role of TRIM29 in CRC and provides a new target for CRC treatment.

## RESULTS

### TRIM29 is upregulated in CRC

**Figure 1:**
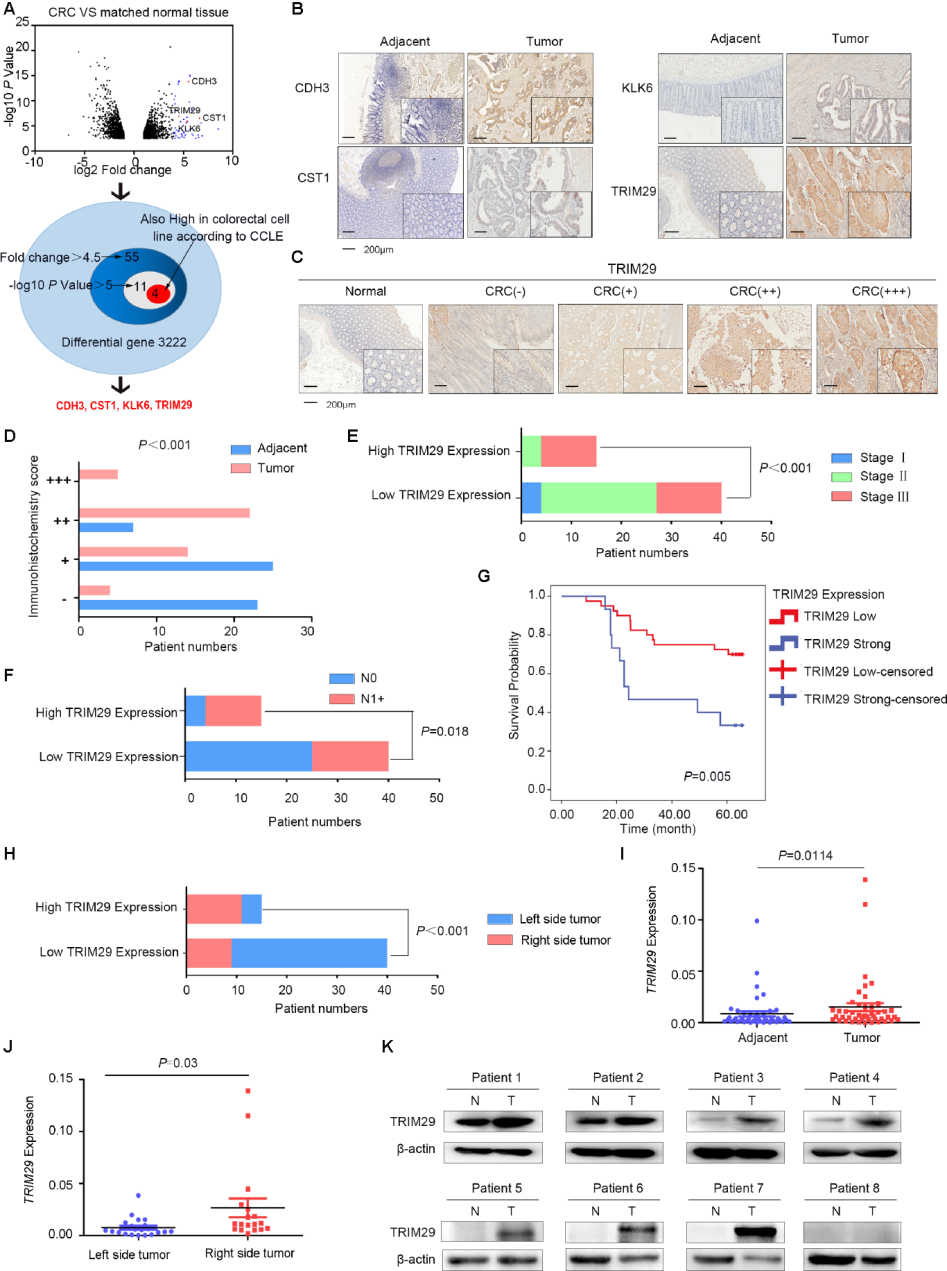
**TRIM29 is upregulated in colorectal cancer.** (**A**) The expression of CDH3, CST1, KLK6 and TRIM29 is upregulated in colorectal cancer (CRC) tissues and cell lines. (**B**) Immunohistochemistry (IHC) results showed that CDH3, CST1, KLK6 and TRIM29 expression was upregulated in CRC. (**C**) Representative IHC images of TRIM29 expression. (**D**) Statistical data graph of patients with different TRIM29 expression levels in CRC tissues and noncancerous samples (*P*<0.0001). (**E**) Statistical data graph of patients with or without lymph node metastasis stratified by TRIM29 expression level (*P*=0.018). (**F**) Statistical data graph of patients at different stages stratified by TRIM29 expression level (*P*<0.0001). (**G**) Kaplan-Meier curves for CRC patients according to TRIM29 expression levels. The statistical analysis was performed using the chi-square test (*P*=0.005). (**H**) IHC results showed that TRIM29 is expressed at much higher levels in RSCC than in LSCC (*P*<0.0001). (**I**) qPCR was used to measure the relative expression levels of TRIM29 in CRC samples and their matched noncancerous samples (*P*<0.05). (**J**) The qPCR results showed that TRIM29 is expressed at much higher levels in RSCC than in LSCC (*P*<0.0001). (**K**) Western blotting was used to detect TRIM29 expression in 8 pairs of samples. The statistical analysis was performed using the chi-square test (**D**–**F**, **H**) and two-tailed Student’s t-test (**I**, **J**). The error bars represent the SEM. Kaplan-Meier method compared with a log-rank test. *P < 0.05, **P < 0.01, ***P < 0.001, ****P < 0.001.

To determine key proteins in CRC, an analysis of our previous mRNA array was performed (Supplementary Figure 1, GEO accession number: GSE110204; comparison of CRC tissues and matched adjacent normal tissues using a 4.5-fold cutoff, log10 *P* value >5 cutoff and high expression in a colorectal cell line according to CCLE; https://portals.broadinstitute.org/ccle). As shown in Figure 1A, four mRNAs that encode KLK6, CDH3, CST1 and TRIM29 were upregulated in CRC.

Immunohistochemistry (IHC) was used to detect KLK6, CDH3, CST1 and TRIM29 protein levels in 55 formalin-fixed, paraffin-embedded CRC tissues and matched adjacent normal tissues. Twenty of these samples were right-sided tumors, and 35 were left-sided tumors. The IHC results revealed that these four candidate proteins were upregulated in CRC to varying degrees (Figure 1B–1D), but only TRIM29 expression was associated with lymph node metastasis (Figure 1E, *P*=0.018), advanced stage (Figure 1F, *P*<0.001) and poor prognosis of CRC (Figure 1G, *P*=0.005). The median overall survival (OS) was 64 months in patients with low TRIM29 expression and 24 months in patients with strong TRIM29 expression; the latter group had shorter OS. More interestingly, TRIM29 is much more highly expressed in RSCC than in LSCC (see Figure 1H, *P*<0.0001). Therefore, in this study, we chose to focus on TRIM29 and elucidated its role in CRC tumorigenesis.

qPCR was used to measure the TRIM29 mRNA level in another cohort of CRC tissues and their normal tissue counterparts from 46 patients; among them, 40 had accurate information on the tumor side: 22 LSCC and 18 RSCC. The qPCR results revealed that TRIM29 expression was significantly higher in tumor tissues than in paired normal tissues (see Figure 1I, *P*<0.05) and was much more highly expressed in RSCC than in LSCC (see Figure 1J, *P*<0.0001). Subsequently, 8 of the 46 samples were subjected to Western blotting. The cohort of CRC tissues showed higher TRIM29 protein expression than that in the normal tissue samples (Figure 1K).

### GATA2 transcriptionally represses TRIM29 expression

**Figure 2:**
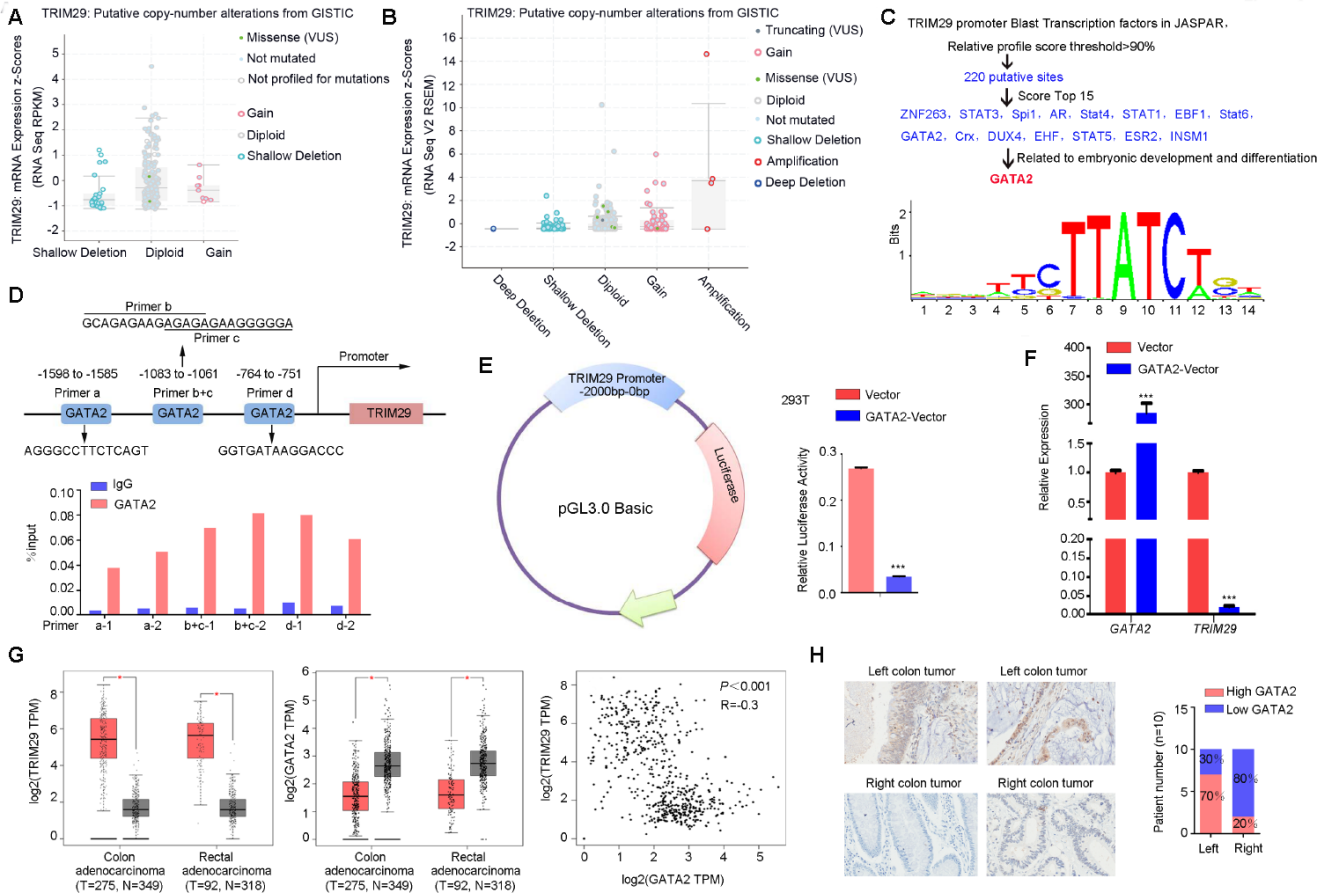
**GATA2 transcriptionally repressed TRIM29 expression.** (**A**, **B**) The low frequencies of mutation (A) (1%) and amplification (**B**) (<1%) for TRIM29 in the TCGA database. (**C**, **D**) Analysis of possible physical associations of GATA2 with regions in the TRIM29 promoter. ChIP assays were performed with GATA2 antibody. IgG served as the negative control. (**E**) Relative luciferase activity in 293T cells transfected with reporter constructs containing the TRIM29 promoter. Luciferase reporter activity was normalized to Renilla luciferase activity. (**F**) The mRNA expression level of TRIM29 in cells transfected with GATA2 plasmid was detected using qPCR. (**G**) GATA2 was negatively correlated with TRIM29. (**H**) Representative images of GATA2 expression in right-sided colorectal cancer (RSCC) and left-sided colorectal cancer (LSCC). RSCC had a higher frequency of loss of GATA2 than did LSCC. The error bars represent the SEM. *P < 0.05, reporter activity was normalized to Renilla luciferase activity. ChIP assay and luciferase reporter assay (**D**–**F**) repeated at least 3 times.

To explore why TRIM29 was highly expressed in CRC, we first determined the gene amplification and mutation status in cohorts from public databases. The results showed a low frequency of amplification and mutation for TRIM29 (Figure 2A, 2B), so we considered that TRIM29 was regulated by a transcription factor. Given that TRIM29 was particularly highly expressed in RSCC, which has different embryonic origins than LSCC, we expected to find a transcription factor related to embryonic development. The JASPAR database was used to blast the transcription factors that could recognize sequences in the TRIM29 promoter region. A total of 220 potential sites were found by using a 90% relative profile score threshold cutoff. The top 15 sites were selected, and their functions as described in the literature were reviewed [10–22]. Of the predicted transcription factors, GATA2 became our primary focus because of its regulatory transcription function in mesoderm [22–28]. (Figure 2C). The matching sequence and possible physical associations with regions in the TRIM29 promoter are shown in Figure 2D. ChIP assays revealed that GATA2 effectively bound to the three corresponding binding elements in the TRIM29 promoter; qPCR using two primer pairs was applied for each binding element (Figure 2D). Next, we constructed a luciferase reporter plasmid containing the -2000 bp ~ 0 bp region of the TRIM29 promoter sequence. Luciferase reporter assays revealed that luciferase activity in the 293T cell line, driven by the TRIM29 promoter sequence, was significantly decreased when GATA2 was overexpressed (Figure 2E).

We overexpressed GATA2, after which the mRNA expression levels of TRIM29 were obviously downregulated (Figure 2F). The correlation of mRNA expression levels between GATA2 and TRIM29 was assessed using data from the GEPIA public database. The results showed that GATA2 was negatively correlated with TRIM29 (Figure 2G), which corresponded to the inhibitory function of GATA2 on transcription. Next, IHC was performed in 20 formalin-fixed, paraffin-embedded tumor tissues and their matched adjacent normal tissues (10 LSCC and 10 RSCC samples, which were previously used to measure TRIM29 expression in Figure 1) to examine the GATA2 expression levels. The results revealed that compared to LSCC, RSCC had a higher frequency of loss of GATA2 expression (Figure 2H).

### TRIM29 promotes the malignant CRC phenotype *in vitro*

**Figure 3:**
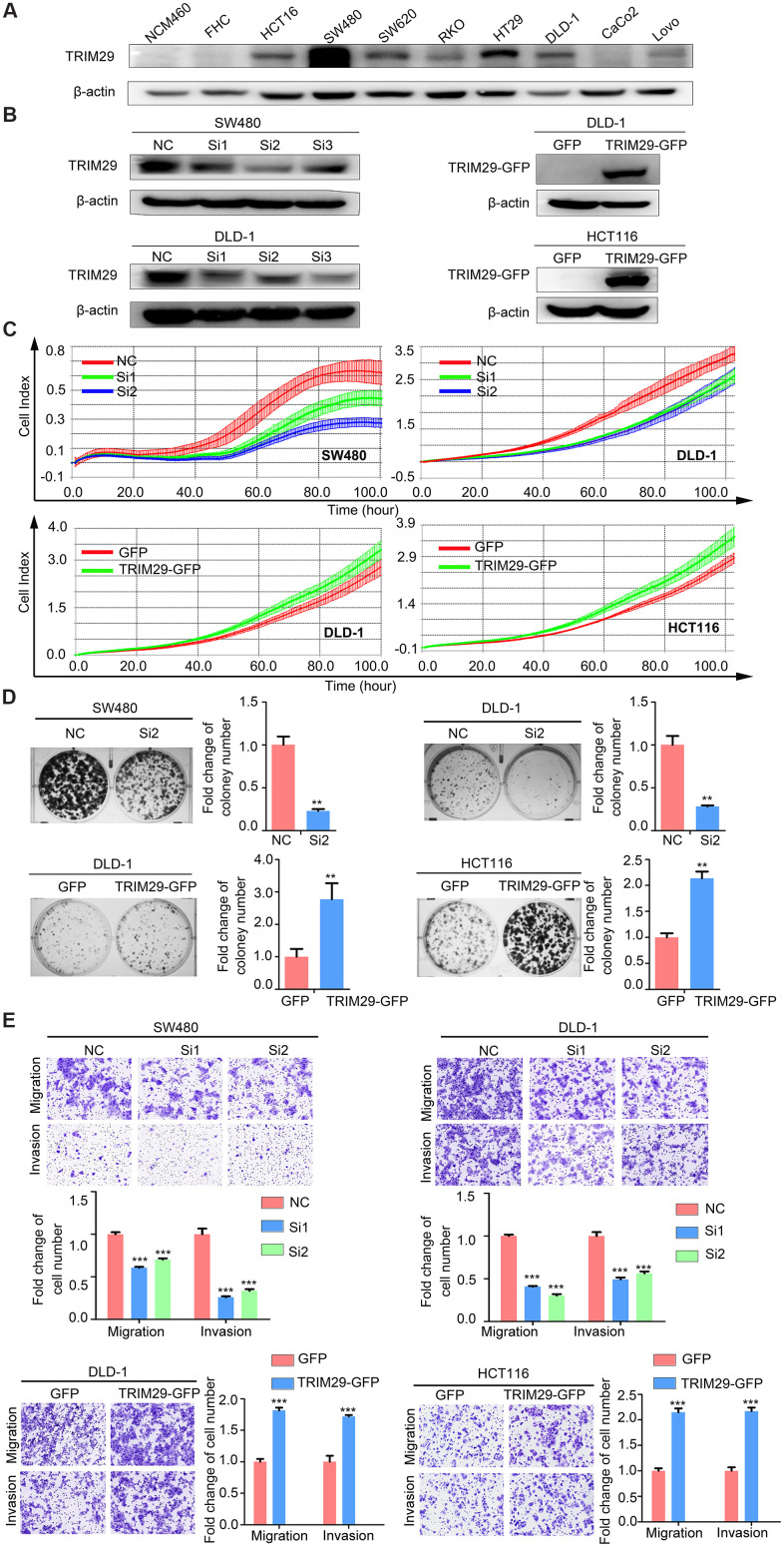
**TRIM29 promotes the malignant phenotype of CRC *in vitro*.** (**A**) Western blotting was used to analyze the level of TRIM29 in CRC cell lines. (**B**) The efficiency of TRIM29 knockdown using siRNA in SW480 and DLD-1 cells and TRIM29 overexpression in DLD-1 and HCT116 cells. (**C**–**E**) SW480 and DLD-1 cells were transfected with siRNA, while HCT116 and DLD-1 cells were transfected with the TRIM29 overexpression plasmid. (**C**) After transfection for 24 h, an RTCA-MP proliferation assay was performed to measure cell proliferation. (**D**) Colony formation assay. (**E**) Transwell assays were performed to measure cell migration and invasion. The error bars represent the SEM. The statistical analysis was performed using two-tailed Student’s t-test. **P < 0.01, ***P < 0.001, ****P < 0.0001. Every experiment repeated at least 3 times.

TRIM29 is upregulated in CRC, especially in RSCC. Since there is no specific cell line model for RSCC and LRCC, we do not discuss the different functions of TRIM29 in RSCC and LRCC but rather study its common role in CRC in this report. We first analyzed the levels of TRIM29 in a panel of CRC cell lines. Among immortalized colon cell lines tested, SW480 cells had the highest expression of TRIM29, followed by the HT29 cell line. The SW620, DLD-1, HCT116, and RKO cell lines showed slightly lower expression levels (Figure 3A). Therefore, the SW480 and DLD-1 cell lines, which exhibited high and moderate expression levels of TRIM29, were transfected with siRNA targeting TRIM29. The DLD-1 and HCT116 cell lines, with moderate and lower levels of TRIM29 expression, were transfected with a pGV230-TRIM29 overexpression plasmid. These cell lines were used to investigate the phenotypic effects in CRC; the transfection efficiencies are shown in Figure 3B. After transfection, we examined the ability of cell proliferation, cloning, migration and invasion (Figure 3C–3E). The results showed that cells transfected with TRIM29-targeted siRNA had significantly lower rates of cell proliferation than those transfected with the NC siRNA. Similarly, the cellular cloning ability was inhibited in cells transfected with TRIM29-targeted siRNA compared to that in cells transfected with NC siRNA. In addition, transwell assays revealed that compared to the NC cells, cells transfected with TRIM29-targeted siRNAs exhibited significantly weakened migration and invasion abilities. In contrast, compared to the pGV230-vector, the TRIM29 overexpression vector significantly promoted CRC cell proliferation, clone ability, migration and invasion. These results, along with the above findings, suggest that TRIM29 is an oncogene for colorectal cancer.

### TRIM29 promotes the growth and metastasis of CRC *in vivo*

**Figure 4:**
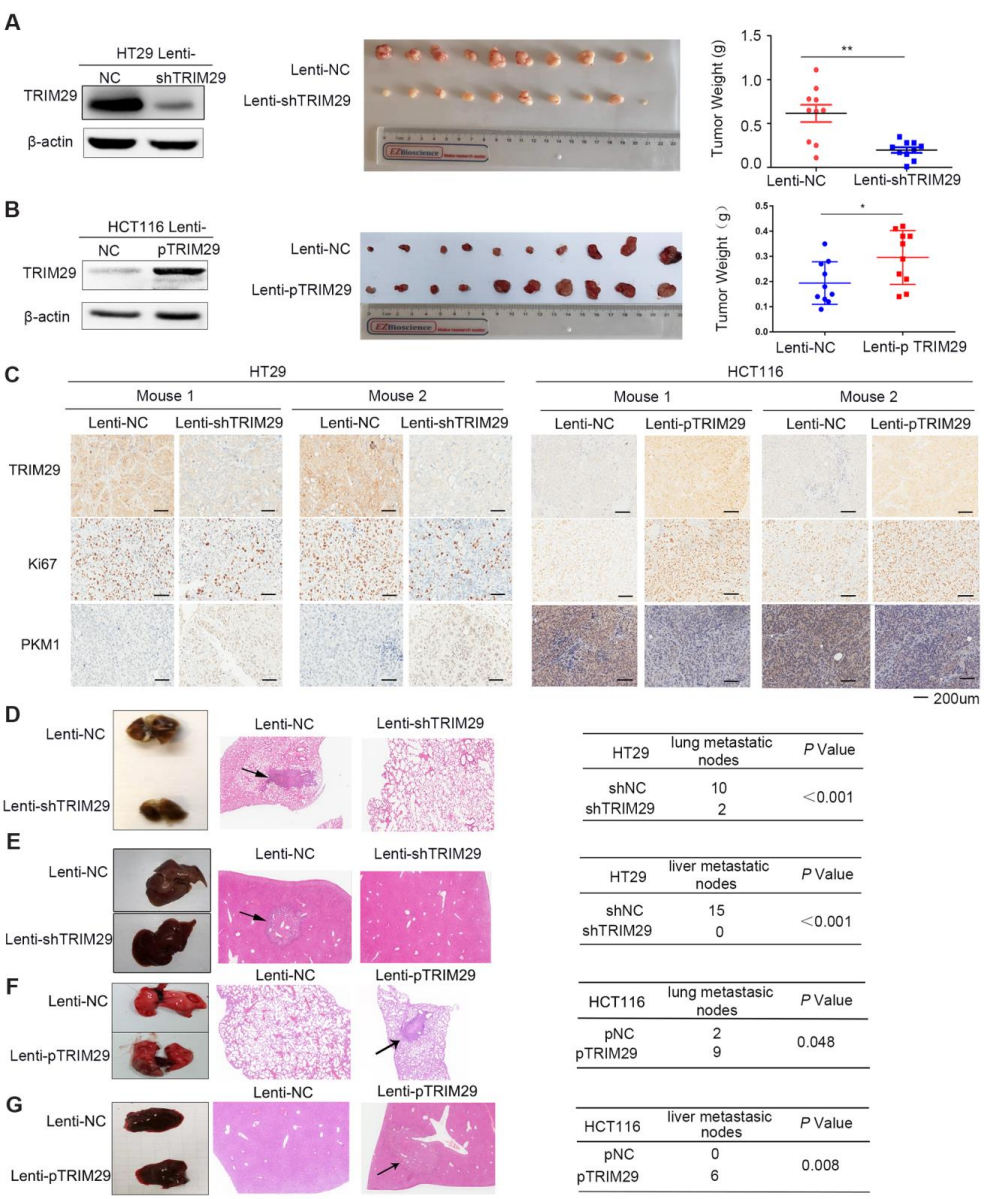
**TRIM29 promotes the growth and metastasis of CRC *in vivo*.** (**A**, **B**) Mice were subcutaneously injected with HT29 cells (**A**) or HCT116 cells (**B**) in both side of the top back (lenti-NC in the left and lenti-shTRIM29/lenti-pTRIM29 in the right). Tumor weight of both lenti-NC and lenti-shTRIM29/lenti-pTRIM29 cell-derived tumors in nude mice was measured two weeks later (n=10). (**C**) Representative IHC staining of TRIM29, Ki67 and PKM1 in two mice with HT29 and HCT116 xenografts derived from lenti-NC and lenti-shTRIM29/lenti-pTRIM29 cells. (**D**–**G**) Mice were injected with HT29 or HCT116 cells (2*10^6^ cells in 0.1 ml) via tail vein; lenti-NC group (n=5), lenti-shTRIM29/lenti-pTRIM29 group (n=5). (**D**, **F**) Rate of lung metastasis after tail injection. (**E**, **G**) Rate of liver metastasis after tail injection. The statistical analysis was performed using two-tailed Student’s t-test. *P < 0.05, **P < 0.01, ***P < 0.001.

To assess the effect of TRIM29 on CRC *in vivo*, one pooled sets of HT29 cells were stably infected with negative control (lenti-NC) or shTRIM29 lentiviral vectors (lenti-shTRIM29) and one pooled sets of HCT116 cells were stably infected with negative control (lenti-NC) or pTRIM29 lentiviral vectors (lenti-pTRIM29) were constructed, and TRIM29 expression was examined by Western blotting. As expected, compared to the lenti-NC cells, the lenti-shTRIM29 cells expressed much lower levels of TRIM29 and the lenti-pTRIM29 cells expressed much higher levels of TRIM29 (Figure 4A, 4B). In addition, the aggressive phenotype was dampened in these stably infected cells (Supplementary Figure 2A, 2B). Compared with tumors in the lenti-NC group, tumors in the lenti-shTRIM29 group grew more slowly, which resulted in smaller tumor masses, and exhibited lower levels of TRIM29 and Ki67, and tumors in the lenti-pTRIM29 group got the opposite results. (Figure 4A–4C).

Two pools of stably infected HT29 cells were separately injected into the tail veins of 5 nude mice. After two months, the presence of lung and liver metastases was analyzed (Figure 4D, 4E). Four of the five control group mice developed lung metastases; by contrast, only two of the five shTRIM29 mice developed lung metastases. The lenti-NC group had 10 lung metastatic nodes, whereas the lenti-shTRIM29 group had 2 lung metastatic nodes (Figure 4D). Furthermore, four of the five lenti-NC group mice presented liver metastases; by contrast, only one of the five lenti-shTRIM29 group mice developed liver metastases. The lenti-NC group had 15 liver metastatic nodes, whereas the lenti-shTRIM29 group had 0 liver metastatic nodes (Figure 4E). These results revealed that decreasing TRIM29 obviously weakens the growth and metastatic ability of CRC *in vivo*.

### TRIM29 physically interacts with PKM1

**Figure 5:**
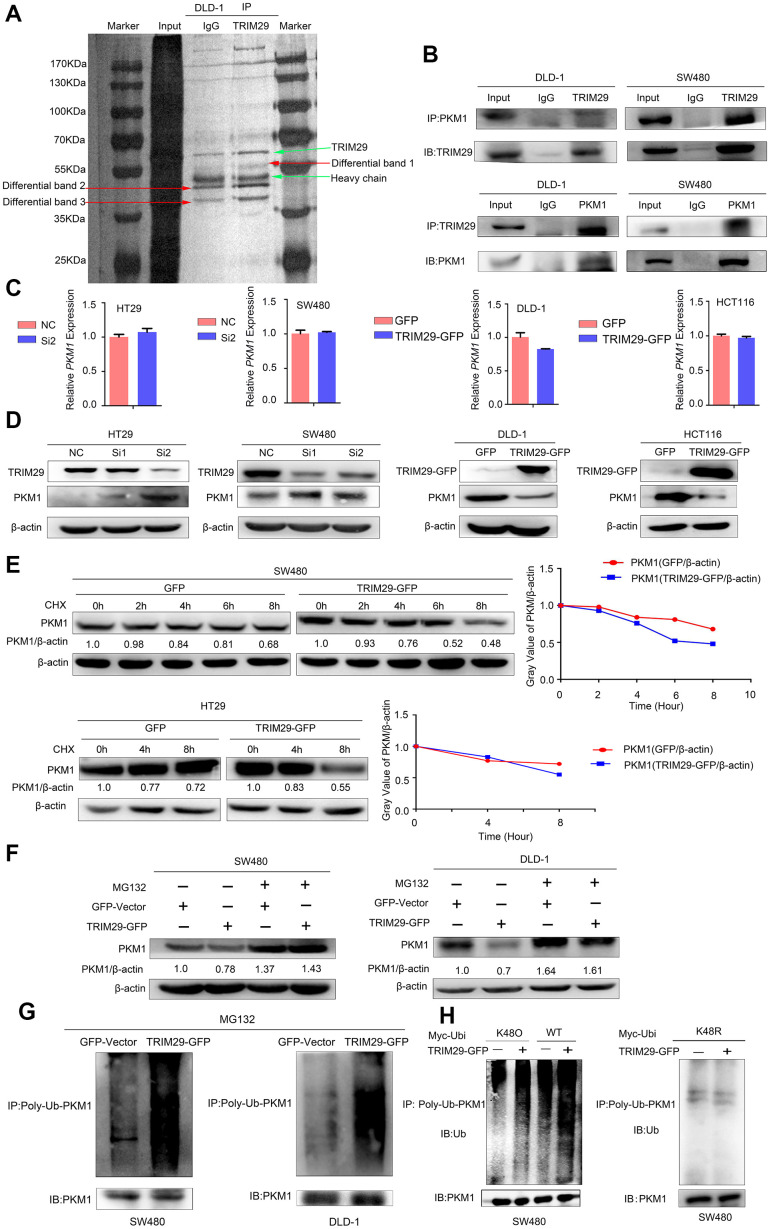
**TRIM29 negatively regulates PKM1 by promoting its degradation via ubiquitination.** (**A**) A coimmunoprecipitation (Co-IP) assay was performed to identify the proteins in DLD-1 cells that interact with TRIM29. (**B**) The Co-IP experiments showed that TRIM29 interacted with PKM1 in DLD-1 and SW480 cell lines. (**C**, **D**) qPCR and Western blotting were used to measure the mRNA and protein expression levels of PKM1 after overexpressing and knocking down TRIM29. (**E**) Cells were transfected with vector and TRIM29 plasmid and followed by 100 μM cycloheximide (CHX). The expression levels of PKM1 were examined by Western blotting every 2 h in SW480 cells and every 4 h in DLD-1 cells. The degradation rate of PKM1 is shown in the right chart. (**F**–**G**) Cells were transfected with vector or TRIM29 plasmid for 36 h and treated with 10 μM MG132 (a proteasome inhibitor) for an additional 12 h. (**F**) PKM1 protein levels were examined in SW480 and DLD-1 cells. (**G**) Co-IP assays were performed. The levels of polyubiquitinated (Poly-Ub) PKM1 proteins was examined by Western blot with anti-ubiquitin antibody in SW480 and DLD-1 cells. (**H**) The levels of wild type, K48O and K48R polyubiquitinated (Poly-Ub) PKM1 proteins was examined by Western blot with anti-ubiquitin antibody in SW480 cells. The statistical analysis was performed using Kendal’s tau-b test. *P < 0.05, **P < 0.01, ***P < 0.001. Every experiment repeated at least 3 times.

To further investigate the mechanism by which TRIM29 affects CRC progression, an immunoprecipitation (IP) combined mass spectrometry (MS) assay was used to obtain the downstream molecular mechanism. Three differential electrophoretic bands between 35 kDa and 70 kDa in the two groups were found after silver staining (Figure 5A). Five proteins were detected in the corresponding region according to the molecular weight (Table 1). The function of 5'-nucleotidase isoform 2 preproprotein (NT5E) in cancer is unclear. The primary histopathological classification of colon cancer is adenocarcinoma, but Serpin B3 is a molecular marker specific to squamous cancer. Therefore, we did not choose either of these proteins for further study.

**Table 1:** Identification of proteins interacting with TRIM29 by mass spectrometry.

**Description**	**Coverage**		**Molecular Weight [kDa]**
	**IgG**	**IP**	
Serpin B3 [Homo sapiens]	4.87	33.85	44.54
5'-nucleotidase isoform 2 preproprotein [Homo sapiens]	0	8.21	57.91
annexin A2 isoform 2 [Homo sapiens]	0	10.91	38.58
L-lactate dehydrogenase A chain [Homo sapiens]	0	5.72	36.67
pyruvate kinase M1 [Homo sapiens]	0	2.84	49.87

The enrichment statistics of the KEGG pathway analysis for our mRNA array (GEO accession number: GSE110204, for the comparison of CRC tissues and matched adjacent normal tissues) showed that DEGs were enriched in cellular processes, environmental information processing, genetic information processing, human diseases, metabolism, and organismal systems (Supplementary Figure 3A, 3B) [9]. Annexin A2 isoform 2 (ANXA2) has been reported to play an important role in tumor migration and invasion, and L-lactate dehydrogenase A chain (LDHA) and PKM1 have both been reported to be closely related to tumor glycometabolism. Since TRIM29 is a ubiquitin E3 ligase, the most likely mechanism of TRIM29 is mediating the ubiquitination of downstream genes. In our study, TRIM29 expression is enhanced in CRC, so the expression of its candidate downstream gene should be decreased in CRC. The TCGA and GTEx datasets (GEPIA, http://gepia.cancer-pku.cn/index.html) revealed that the expression levels of AXAN2 and LDHA were upregulated in colon adenocarcinoma and rectal adenocarcinoma (Supplementary Figure 3C). Overall, we selected PKM1, which plays a key role in tumor glycometabolism, as the focus of subsequent experiments.

The relationships between TRIM29 and PKM1 were verified by using reciprocal coimmunoprecipitation (Co-IP) experiments carried out in SW480 and DLD-1 cells, respectively. The results confirmed the interaction between endogenous TRIM29 and PKM1 (Figure 5B).

### TRIM29 regulates glycolytic metabolism by promoting the ubiquitination degradation of PKM1

To understand how TRIM29 regulates PKM1, we used qPCR and Western blotting to measure the mRNA and protein expression levels, respectively, of PKM1 after overexpressing and knocking down TRIM29. The results showed that the mRNA level of PKM1 did not change significantly whereas PKM1 protein levels were negatively correlated with TRIM29 expression (Figure 5C, 5D).

We suspected that TRIM29 might regulate the expression of PKM1 at the translational or posttranslational level. Specifically, since TRIM29 is a ubiquitin E3 ligase, we investigated whether TRIM29 regulates the PKM1 protein level by mediating the ubiquitination status of PKM1. A cycloheximide (CHX) pulse-chase assay was performed to examine the function of TRIM29 in PKM1 stability. Subsequently, we observed that overexpressing TRIM29 could decrease PKM1 protein levels and shorten its half-life (Figure 5E). The loss of PKM1 induced by TRIM29 overexpression was blocked with the proteasome inhibitor MG132 (Figure 5F), which suggests that TRIM29 degrades PKM1. The ubiquitination levels of PKM1 were both enhanced in the SW480 and DLD-1 cell lines transfected with TRIM29-GFP compared with those transfected with the GV230 vector, which suggests that TRIM29 overexpression allowed more PKM1 to enter the degradation process (Figure 5G). These findings indicate that TRIM29 regulates PKM1 by promoting its degradation via ubiquitination. For protein ubiquitination, protein degradation is mediated by K48 ubiquitination, so we tested if TRIM29 induces the K48-mediated ubiquitination of PKM1. We transfected wild type ubiquitin(WT), or ubiquitin mutants retaining K48 lysine residue (K48O) or retaining all but K48 lysine residues (K48R) in the presence or absence of TRIM29 followed by ubiquitination analysis by using SW480 cell line. The results showed that TRIM29 attach K48O-linked but not K48R-linked polyubiquitin chains to PKM1 in cells (Figure 5H). So we consider that TRIM29 induces the K48-mediated ubiquitination of PKM1.

**Figure 6:**
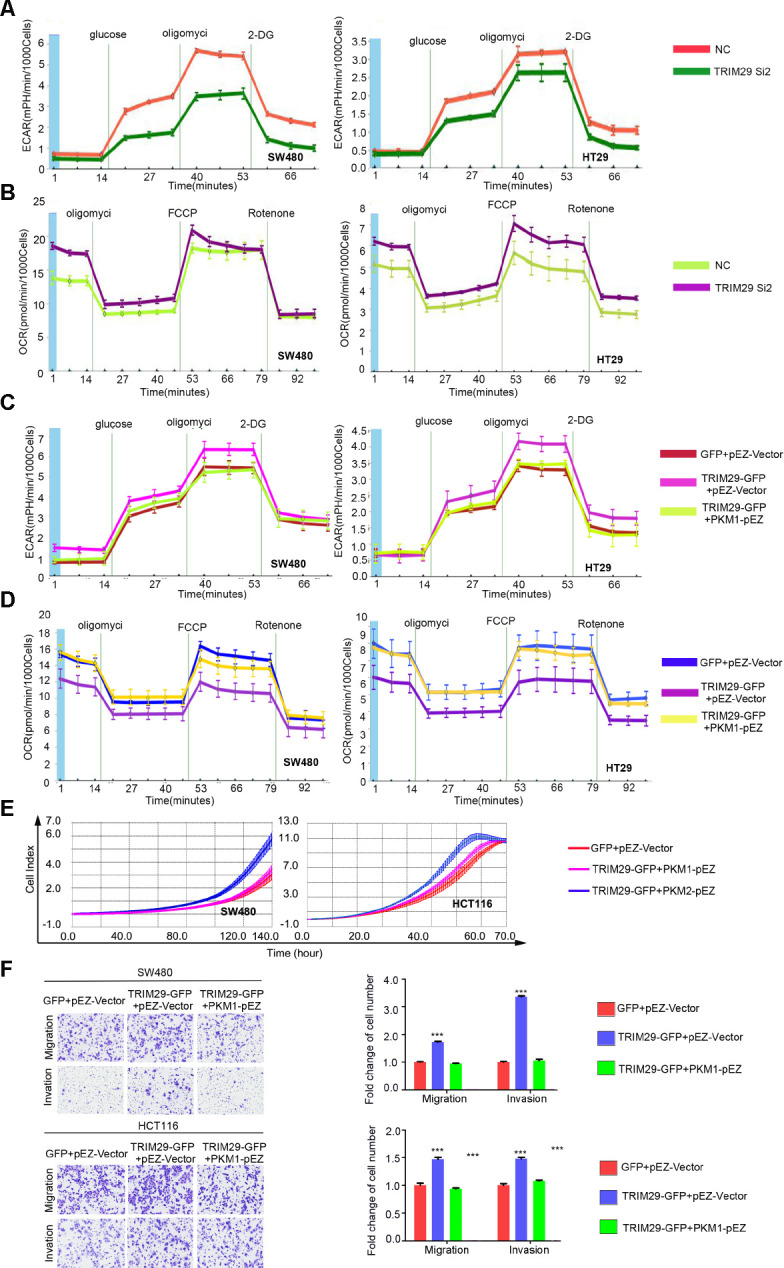
**TRIM29 regulates glycolytic metabolism and the aggressive phenotype in CRC by regulating PKM1.** (**A**, **B**) After transfection with NC or Si2 in SW480 and HT29 cell lines, (**A**) The extracellular acidification rate (ECAR) and (**B**) the oxygen consumption rate (OCR) of cells was measured. (**C**–**F**) Cells transfected with GV230 vector plus Ez-M98 vector, TRIM29 plasmid plus Ez-M98 vector, TRIM29 plasmid plus PKM1 plasmid. (**C**) The ECAR and (**D**) OCR of cells were measured. (**E**) Growth was determined using the RTCA-MP system. (**F**) Migration and invasion were measured using a transwell assay. The statistical analysis was performed using the two-tailed Student’s t-test. **P < 0.01, ***P < 0.001. Every experiment repeated at least 3 times.

Because the main function of PKM1 is to regulate glycolytic metabolism, we next tested whether altered TRIM29 levels directly influence glycolytic metabolism in CRC cell lines by measuring the extracellular acidification rate (ECAR) and oxygen consumption rate (OCR). Indeed, knocking down TRIM29 in SW480 and HT29 cells significantly increased the OCR and reduced the ECAR compared those in the respective control cells (Figure 6A, 6B). To confirm the function of PKM1 in glycolytic metabolism, the ECAR and OCR were measured in SW480 and HCT116 cells transfected with PKM1 plasmids. Unsurprisingly, compared with the control, overexpression of PKM1 significantly increased the OCR and decreased the ECAR (Supplementary Figure 4A, 4B).

### TRIM29 promotes malignant CRC cell phenotypes by decreasing PKM1 expression

To demonstrate that the role of TRIM29 in CRC is dependent on glycolytic metabolism via regulation of PKM1, rescue experiments were performed in SW480 and HT29 cells. The results suggest that when TRIM29 was overexpressed, the OCR decreased, but the ECAR and malignant CRC cell phenotypes increased dramatically. The OCR, ECAR and malignant CRC cell phenotypes were partially rescued by ectopic expression of PKM1 in the TRIM29-GFP plasmid-treated cells (Figure 6C–6F).

### TRIM29 protein negatively correlates with GATA2 protein and PKM1 protein in CRC

**Figure 7:**
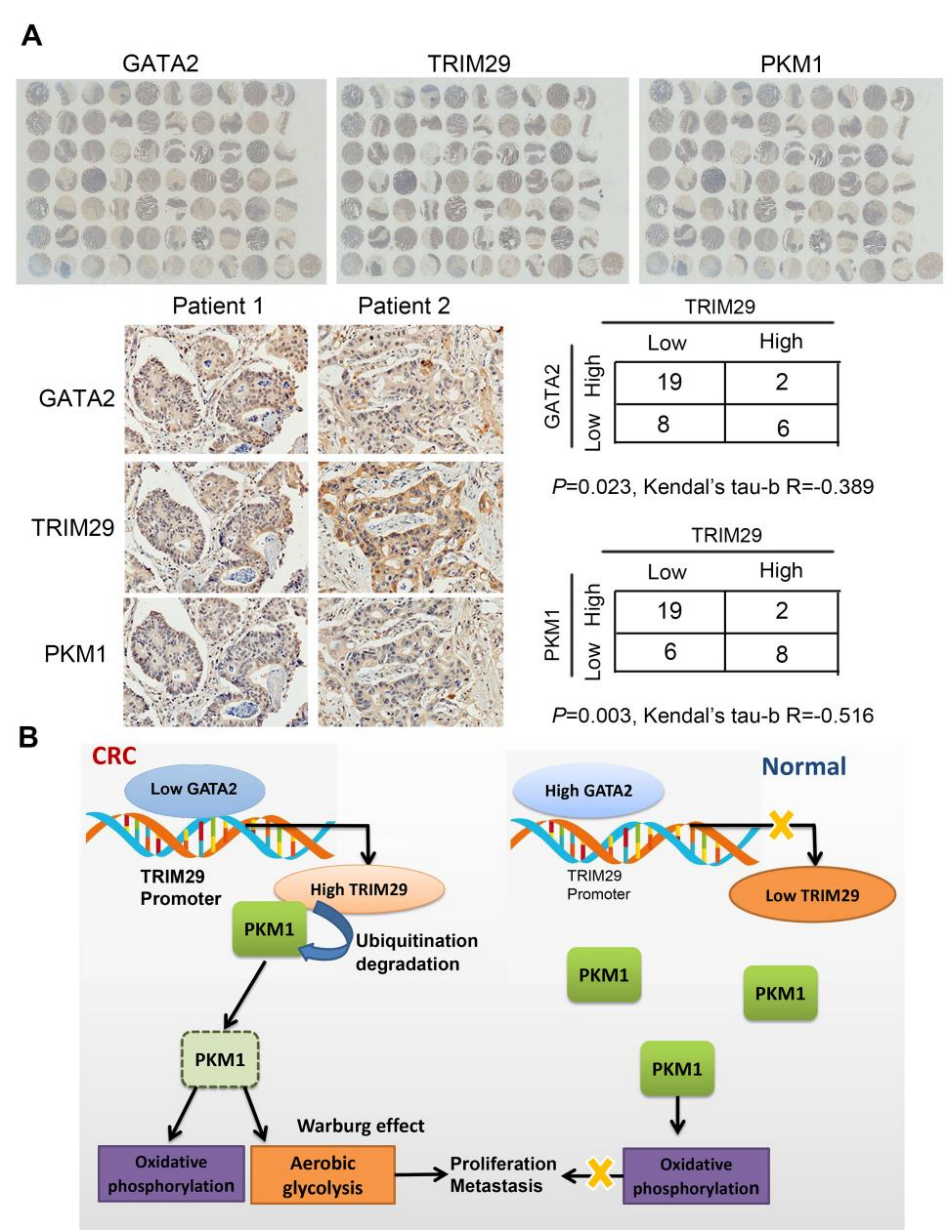
**TRIM29 protein negatively correlates with GATA2 protein and PKM1 protein in CRC.** (**A**) IHC results of TRIM29, GATA2 and PKM1 in the tissue array. (**B**) Model of the GATA2-TRIM29-PKM1 axis in CRC. The statistical analysis was performed using the Kendual’s tau-b test. *P < 0.05, **P < 0.01.

To further confirm the correlation between TRIM29 protein levels and PKM1/GATA2 protein levels, three duplicates of the same tissue microarrays were used to test GATA2, TRIM29, and PKM1 expression. The results revealed that TRIM29 protein levels negatively correlated with GATA2/PKM1 protein levels (Figure 7A). Model of the GATA2-TRIM29-PKM1 axis in CRC was shown in Figure 7B.

## DISCUSSION

The complex process of CRC pathogenesis, which involves multistep molecular pathways initiated by genetic and epigenetic events, makes identifying prognostic biomarkers and developing targeted therapies for CRC difficult. Recently, the influence of tumor site on the treatment and prognosis of CRC has also attracted widespread attention. Splenic curvature is currently used clinically to distinguish RSCC and LSCC because it is the boundary of their different embryonic origins. The clinical manifestations, intestinal flora, treatment response and prognosis of RSCC and LSCC are completely different. However, accurate biomarkers and mechanistic characteristics explaining the differences in LSCC and RSCC are still unknown. Accordingly, it is necessary to find targets specific for LSCC and RSCC. Our study confirmed that TRIM29 is highly expressed in CRC, especially in RSCC. TRIM29 functions as a tumor-promoting factor in CRC and can also explain the difference between LSCC and RSCC to some extent.

Generally, there are three major mechanisms involved in the regulation of gene expression: genetic level (amplification or deletion), epigenetic (histone modifications and transcription factor regulation) and posttranscriptional translation level (noncoding RNAs, etc.). The TRIM29 locus has a low frequency of copy number alterations in CRC. Previous studies confirmed that TRIM29 is regulated by varying miRNAs, such as miR-185 in gastric cancer and miR-621 in bladder cancer [29, 30]. TRIM29 shows higher expression in RSCC than in LSCC. The RSCC originates from the mesoderm; and the ectopic expression of TRIM29 in CRC is probably regulated by specific mesoderm-related genes. We have not identified any mesoderm-related miRNAs, but a comprehensive analysis and our experimental results revealed that TRIM29 is negatively regulated by the transcription factor GATA2, which plays an important role during mesodermal diversification [22]. Our work is the first to characterize abnormal TRIM29 expression from a developmental perspective.

The ubiquitin E3 ligase TRIM29 was initially considered an inflammatory indicator [8]. It has been reported that TRIM29 controls innate immunity against viral and bacterial respiratory infections [31]. Recent studies verified TRIM29 as an oncogene in many kinds of cancers, such as bladder, pancreatic, and gastric cancer [29, 32–34]**.** It’s reported that TRIM29 ubiquitinates and degrades the TGF-β-activated kinase 1 binding protein 2 (TAB2), a key adaptor protein in IFN-γ production by NK cells which suggested that TRIM29 as a negative regulator of NK cell functions [35]. It’s also reported that the dysfunction of Natural Killer Cells can lead to the development of tumor [36]. So the high expression of TRIM29 disrupts the function of NK cells, which may contribute to the development of cancer. Our study confirmed that TRIM29 regulates glycometabolism through the ubiquitination of PKM1 isoform. TRIM29 degrades PKM1 and causes the glucose metabolism of colorectal cancer cells flowing from oxidative phosphorylation to glycolysis.

It has been verified that advanced stage in CRC is related to glycolysis [37–39], which can be used as a target for developing therapeutics for the treatment of CRC [37, 39]. Pyruvate kinase is an enzyme that catalyzes the conversion of phosphoenolpyruvate and ADP to pyruvate and ATP in glycolysis and plays a role in regulating cell metabolism. There are four mammalian pyruvate kinase isoforms (PKM1, PKM2, PKR, and PKL). PKM1 is mainly expressed in muscle, brain and terminally differentiated cells with high energy demand, while PKM2 is mainly expressed in embryonic cells, stem cells and tumor cells with high anabolic demand. The embryonic isoform PKM2 is almost universally re-expressed in cancers and promotes aerobic glycolysis, whereas the adult isoform PKM1 promotes anaerobic glycolysis. Glycolysis capacity increased significantly when PKM2 occupied the main position. Decreased expression of PKM1 and re-expression of PKM2 explains the Warburg effect seen in cancer cells and ensures maximal tumorigenicity. In this report, we found that TRIM29 regulates PKM1 in CRC via ubiquitination. We demonstrated that increased PKM1 expression (PKM2 occupying the secondary position) leads to reduced malignant behavior, weakened glycolysis and enhanced oxidative phosphorylation. By contrast, a decrease in PKM1 (PKM2 occupying the lead position) resulted in stronger malignant behavior, enhanced glycolysis and weakened oxidative phosphorylation.

Taken together, our data indicate that the TRIM29-PKM1 axis promotes an aggressive phenotype in tumors by enhancing glycolysis and weakening oxidative phosphorylation. TRIM29 is a potential target for coordinating glycolysis in CRC; however, owing to the lack of specific cell line models for RSCC and LRCC, we could not perform more in-depth research on the function of TRIM29 in RSCC. Further studies are required to delineate the specific role of TRIM29 in RSCC.

## MATERIALS AND METHODS

### Ethics statement

This study was approved by the Ethical Review Boards of Hebei Medical University Fourth Affiliated Hospital and the committee’s reference number was 2017MEC115. Investigation has been conducted in accordance with the ethical standards and according to the Declaration of Helsinki and according to national and international guidelines and has been approved by the authors' institutional review board.

### Cell culture

The CRC cell lines HCT116, SW480, SW620, DLD-1, HT-29, RKO, HCT-8, and Lovo and the immortalized colon epithelial cell lines NCM460 and FHC were cultured in RPMI 1640 (Gibco) supplemented with 10% fetal bovine serum (FBS) and antibiotics. HT-29 cells were cultured in Dulbecco's modified Eagle's medium (DMEM) supplemented with 10% FBS and antibiotics. All of these cells were maintained at 37° C with 5% CO_2_.

### Human colorectal cancer tissue samples

A total of 46 pairs of fresh-frozen human CRC tissues and their matched adjacent normal tissues were obtained from the 4^th^ Hospital of Hebei Medical University. All samples were identified as either colorectal adenocarcinoma tissues or normal colorectal tissues by pathological examination.

### Paraffin sectioning, tissue microarray and immunohistochemical staining

Paraffin sections of human CRC and matched adjacent normal tissues were collected from 55 patients at the 4^th^ Hospital of Hebei Medical University. The three replicates of the same tissue microarray were provided by Anelabio. The tissue specimens and tissue microarrays were deparaffinized in xylene and dehydrated in a graded series of ethanol. Then, endogenous peroxidase activity was blocked in 3% H_2_O_2_ solution, at which point antigen retrieval was performed using a 20-min heat-induced antigen retrieval procedure in pH=9.0 TRIS-EDTA buffer (zsbio). Protein expression was measured using a 3,3-diaminobenzidine (DAB) peroxidase substrate (zsbio). The antibodies used were anti-TRIM29 (Signalway Antibody); anti-CDH3, anti-PKM1, anti-PKM2, and anti-CST1 (Proteintech); anti-KLK6 (Bioss) and anti-GATA-2 (Abcam).

### RNA extraction, RT-PCR and quantitative real-time PCR

Total RNA was extracted from fresh-frozen tissues and cell lines with TRIzol reagent (Invitrogen). cDNAs were synthesized with OneScript Plus Reverse Transcriptase (abm). Quantitative real-time PCR (qPCR) was performed with the OneScript Plus cDNA Synthesis kit (abm). Glyceraldehyde 3-phosphate dehydrogenase (GAPDH) was used as an internal normalization reference. The qPCR primers used are listed as follows: TRIM29 forward: AGCATCAGCGACTCTGTGTTG, TRIM29 reverse: GAAGTTGCCTAGTGACTGTCC; PKM1 forward: TGTACCATTGGCCCAGCTTC, PKM reverse: CAGCCACAGGATGTTCTCGT; GATA2 forward: GCCGGGAGTGTGTCAACTG, GATA2 reverse: AGGTGGTGGTTGTCGTCTGA; GAPDH forward: GAGAAGGCTGGGGCTCATTT, GAPDH reverse: AGTGATGGCATGGACTGTGG.

### Western blot analysis

Fresh-frozen tumor samples and cell lines were lysed in RIPA lysis buffer (Applygen, China) containing a protease inhibitor cocktail. Then, 40 μg of protein lysate per sample was separated by SDS–PAGE on an 8% or 10% gel and transferred to polyvinylidene fluoride membranes (Millipore, Billerica, MA, USA). After the membranes were blocked with 5% skim milk for 1 h at 37° C, they was incubated with primary antibodies overnight at 4° C. The membranes were then treated with corresponding secondary antibodies (Promega, USA) and incubation with a chemiluminescent substrate to develop the protein bands. Photographs were taken with an Image Reader LAS-4000 (Fuji Ltd, 120 Japan). The primary antibodies used were anti-TRIM29 (Signalway Antibody), anti-PKM1(Proteintech), anti-β-actin and anti-ubiquitin (Santa Cruz Biotechnology), and anti-GFP (Cell Signaling Technology).

### Cell proliferation assay

The proliferative ability of cancer cells was monitored by using the xCELLigence Real-Time Cell Analyzer (RTCA)-MP system (Acea Biosciences/Roche Applied Science), which can measure cellular growth status in real time. In brief, 50 μl of culture medium was added to each well of an E-Plate 96 (Roche Applied Science) to achieve equilibrium. Cells were incubated in 6-well cell culture plates for 24-36 h, and then cells (HCT116 and DLD-1, 2000 cells; HT-29 and SW480, 4000 cells) in 100 μl of culture medium were seeded into the wells of the E-Plate 96. The E-Plate 96 was placed in an RTCA-MP instrument at 37° C with 5% CO_2_. Changes in the electrical impedance were measured as a cell index that directly reflects cellular proliferation on biocompatible microelectrode-coated surfaces [40]. The cell proliferation index was measured automatically every 15 min, and the recorded curve is presented as the cell index ± s.e.m.

### Transwell migration/invasion assay

Cell migration was assayed in transwell cell culture chambers with 6.5-mm diameter polycarbonate membrane filters with an 8-μm pore size (Neuro Probe). Cells (2 × 10^5^) in 100 μl of medium with 10% FBS were added to the upper chamber, and the lower chamber was filled with 650 μl of culture medium supplemented with 30% FBS. Cells were maintained at 37° C in an atmosphere containing 5% CO_2_. After the indicated times (HCT116, DLD-1 20 h and SW480, HT29 48 h), the nonmigrating cells were removed from the upper surface of the membrane with a cotton swab. The filters were then fixed in methanol for 5 min, stained with Giemsa solution for 10 min, and counted. At least five random microscopic fields (×100) were counted per well, and the mean was calculated. For the transwell invasion assay, the procedure was performed as described above with the upper chamber membrane precoated with 50 μl of a 2.5 mg ml^−1^ solution of Matrigel (Falcon BD).

### Colony formation assay

A total of 1000 transfected cells were seeded into 6-well cell culture plates and incubated at 37° C with 5% CO_2_ for 14 days. Culture plates were fixed with precooled methanol for 20 min and stained with crystal violet for 15 min. Then, the number of colonies was calculated. Finally, the ability to form clones was measured with a Gel Imaging Analysis System (SYNGENE, USA).

### Oligonucleotide transfection

TRIM29 siRNAs were custom designed and provided by GenePharma (Suzhou, China). Cells were transfected with siRNA using Lipofectamine 2000 reagent (Invitrogen) according to the manufacturer’s protocol. Transfected cells were incubated for 24-48 h at 37° C in an atmosphere containing 5% CO_2_. The following sequences were used for the siRNAs: TRIM29-siRNA1: GCGACCCAUCAUCCAGUUUTT (sense), AAACUGGAUGAUGGGUCGCTT (antisense); TRIM29-siRNA2: GCAAGACGAUGGAGCUCUUTT (sense), AAGAGCUCCAUCGUCUUGCTT (antisense); TRIM29-siRNA3: GCUCAAGAUCAUUGAGAUUTT (sense), AAUCUCAAUGAUCUUGAGCTT (antisense).

### Plasmid construction

The TRIM29 overexpression plasmid was constructed with the GV230 (GFP) vector. The GATA2 and PKM1 plasmids were all constructed in the Ez-NEG-M98 vector. The TRIM29 promoter region (-2000 bp ~ 0 bp) was inserted upstream of the firefly luciferase gene in the pGL3.0 basic vector.

### Luciferase reporter assay

The luciferase reporter assay was performed with the Dual-Luciferase Reporter Assay System (Promega) according to the manufacturer’s protocol.

### Histology and IHC staining interpretation

The IHC staining results were assessed independently by two pathologists who were blinded to the patients’ clinical outcomes. A consensus decision was made when there was an interobserver discrepancy. The staining intensity was scored as follows: 0, no staining; 1+, weak staining; 2+, moderate staining; and 3+, strong staining. High IHC expression was defined as a staining intensity of 3+ in over 25% of tumor cells.

### Immunoprecipitation and LC-MS/MS

DLD-1 cell lysates were obtained, and the total protein concentration were measured. In brief, 2 mg of protein lysate, 30 μl of precleared protein A/G beads (Roche, Switzerland) and 1 μg of primary antibody (rabbit IgG or TRIM29) were incubated together at 4° C overnight. Proteins were then separated by SDS–PAGE, and silver staining was performed after gel electrophoresis. The experiments were repeated three times, and a significant band at 58 KDa (which was identified as PKM1/2) was observed compared to the corresponding band in the control group. The following LC-MS/MS procedures to identify proteins from the samples were carried out by Capitalbio Technology (Beijing, China).

Proteome discoverer software (version 1.4) (Thermo Scientific, USA) was used to perform database searches against the Oryctolagus cuniculus database (46601 proteins) using the Sequest algorithms. The following criteria were applied: precursor mass tolerance of 15 ppm and a fragment mass tolerance of 20 mmu. The results were filtered using the following settings: high confident peptides with a global FDR<1% based on a target-decoy approach were included in the results (Table 1).

### Bioinformatics analysis

To perform the biological process (BP) analysis of DEGs from our previous mRNA assay (GEO accession number: GSE110204) [a9], the DAVID (https://david.ncifcrf.gov/home.jsp) (version 6.8) online tool was utilized. Statistical significance was defined as P<0.05. The result was visualized by the R language.

### Coimmunoprecipitation (Co-IP)

Protein lysate supernatants, primary antibody and precleared protein A/G beads (Roche, Switzerland) were incubated at 4° C overnight according to the manufacturer’s instructions. The protein A/G-antibody-antigen complex was concentrated by centrifugation at 2000×g for 1 min and washed six times with 1% NP40. Proteins were then separated by SDS–PAGE and visualized by Western blotting.

### Cycloheximide assay

Cells were plated in 6-well plates and treated with CHX at a concentration of 100 μg/ml for 24 h. Cells were harvested at 2-h intervals, and protein lysates were prepared as described above.

### ChIP assay

Chromatin immunoprecipitation assays were performed using a Pierce™ Magnetic ChIP Kit (Thermo Scientific™). Immunoprecipitation was performed with anti-GATA2 antibodies (Abcam). Specific regions were quantified via qRT-PCR using the primers listed as follows: TRIM29 primer a-1 forward: GTTCCCAGGGGGAGAAGG, TRIM29 primer a-1 reverse: CAAGCCCAGGATGATGCT; TRIM29 primer a-2 forward: GTTCCCAGGGGGAGAAGG, TRIM29 primer a-2 reverse: CAAGCCCAGGATGATGCT; TRIM29 primer b+c-1 forward: TCTTCCTTCCTCCTCTCCAC, TRIM29 primer b+c-1 reverse: CTCACCCTTACCCTCCTGTC; TRIM29 primer b+c-2 forward: TCTTCCTTCCTCCTCTCCAC, TRIM29 primer b+c-2 reverse: TTCTCACCCTTACCCTCCTG; TRIM29 primer d-1 forward: TGGGTGCTGGTTCCCTACGT, TRIM29 primer d-1 reverse: TGAGAGTCCGGGCATCTCTG; TRIM29 primer d-2 forward: GGGTGCTGGTTCCCTACGT, and TRIM29 primer d-2 reverse: GAGAGTCCGGGCATCTCTG.

### Extracellular acidification rate and oxygen consumption rate assays

The ECAR and cellular OCR were tested by using a Seahorse Extracellular Flux Analyzer XF96 (Seahorse Bioscience) following the manufacturer’s instructions to monitor cell metabolic alterations *in vitro*. The ECAR and OCR were measured using the Seahorse XF Glycolysis Stress Test Kit and Seahorse XF Cell Mito Stress Test Kit (Agilent Technologies), respectively. Cells were seeded in an XF96-well plate at a density of 1.5 × 10^4^ per well and allowed to attach overnight. To detect the real-time values, an indicator of net proton loss during glycolysis was used, and cells were incubated with unbuffered medium for 1 h. Then, for the ECAR, 10 mM glucose, 4 μM oligomycin, and 80 mM 2-deoxyglucose were sequentially injected into each well at the indicated time points; for OCR, 4 μM oligomycin, 0.5 μM reversible inhibitor of oxidative phosphorylation FCCP (p-trifluoromethoxy carbonyl cyanide phenylhydrazone), and 0.5 μM mitochondrial complex I inhibitor rotenone plus mitochondrial complex III inhibitor antimycin A (Rote/AA) were sequentially injected. After detection, the cells were dissociated, and cell numbers were counted. ECAR measurements were normalized to cell numbers and reported as mpH/min/1000 cells, and the OCR was reported as pmol/min. Each sample was assayed four times.

### Analysis of *in vivo* tumorigenicity

Four-week-old female BALB/c nude mice were provided by HFK-bio for the *in vivo* tumorigenicity study. The mice were subcutaneously injected with 2×10^6^ cells in 0.1 ml into their upper backs. In addition, mice received the same injection via tail vein.

### Study approval

This work was approved by the Cancer Hospital, the Chinese Academy of Medical Sciences, the National GCP Center for Anticancer Drugs, and the Independent Ethics Committee. The collection of all of the human samples used in the experiments was approved by the 4^th^ Hospital of Hebei Medical University. Informed consent was obtained from all patients. All experiments involving mice were approved by the Cancer Hospital, the Chinese Academy of Medical Sciences, and the Experimental Animal Ethics Committee and followed Institutional Animal Welfare Guidelines.

### Statistical analysis

Data analysis was performed with the SPSS 17.0 software package. The data are presented as the means ± SEM. The differences between groups were assessed using 2-tailed Student’s t-test with GraphPad Prism 5 software. The correlation between GATA2 and TRIM29 expression and between TRIM29 and PKM1 expression determined via IHC was calculated using Kendall’s tau-b rank correlation analysis. Estimates of survival rates were obtained using the Kaplan-Meier method and compared with a log-rank test. For all analyses, differences were considered significant when P was less than 0.05. *P < 0.05, **P < 0.01, ***P < 0.001. All *in vitro* experiments were repeated at least 3 times unless stated otherwise.

**Availability of data and materials**

The datasets used and/or analyzed during the current study are available from the corresponding author on reasonable request.

## Supplementary Material

Supplementary Figures
