# Supplementary material for: Transcriptional dysregulation of TRIM29 promotes colorectal cancer carcinogenesis via pyruvate kinase-mediated glucose metabolism
Source: Aging (Albany NY). 2021 Jan 20;13(4):5034–54. doi: 10.18632/aging.202414 (PMC7950264; doi:10.18632/aging.202414)
Supplement: Supplementary Figures [file aging-13-202414-s001.pdf]

SUPPLEMENTARY FIGURES

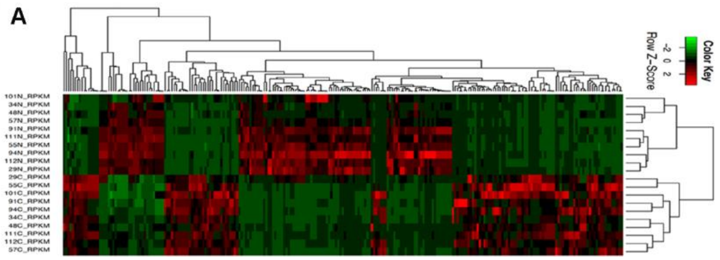

Supplementary Figure 1. (**A**) Heat map of the mRNA profile showing DEGs in CRC.

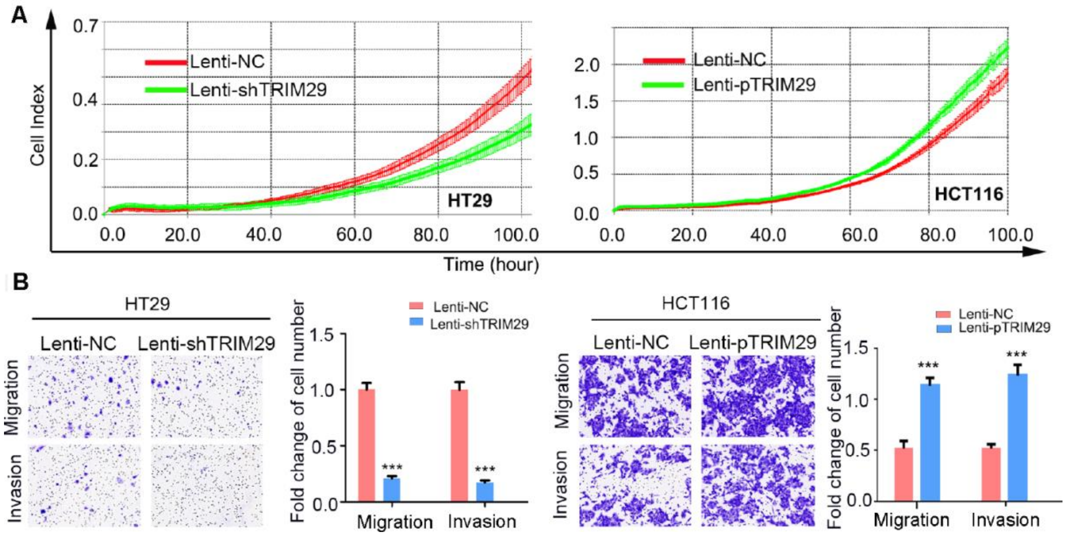

Supplementary Figure 2. HT29 cells were stably infected with lenti-NC or lenti-shTRIM29 and HCT116 cells were stably infected with lenti-NC or lenti-pTRIM29. (**A**) RTCA-MP was performed. (**B**) Transwell assays were performed. The statistical analysis was performed using the two-tailed Student's t-test. ***P* < 0.01, ****P* < 0.001.

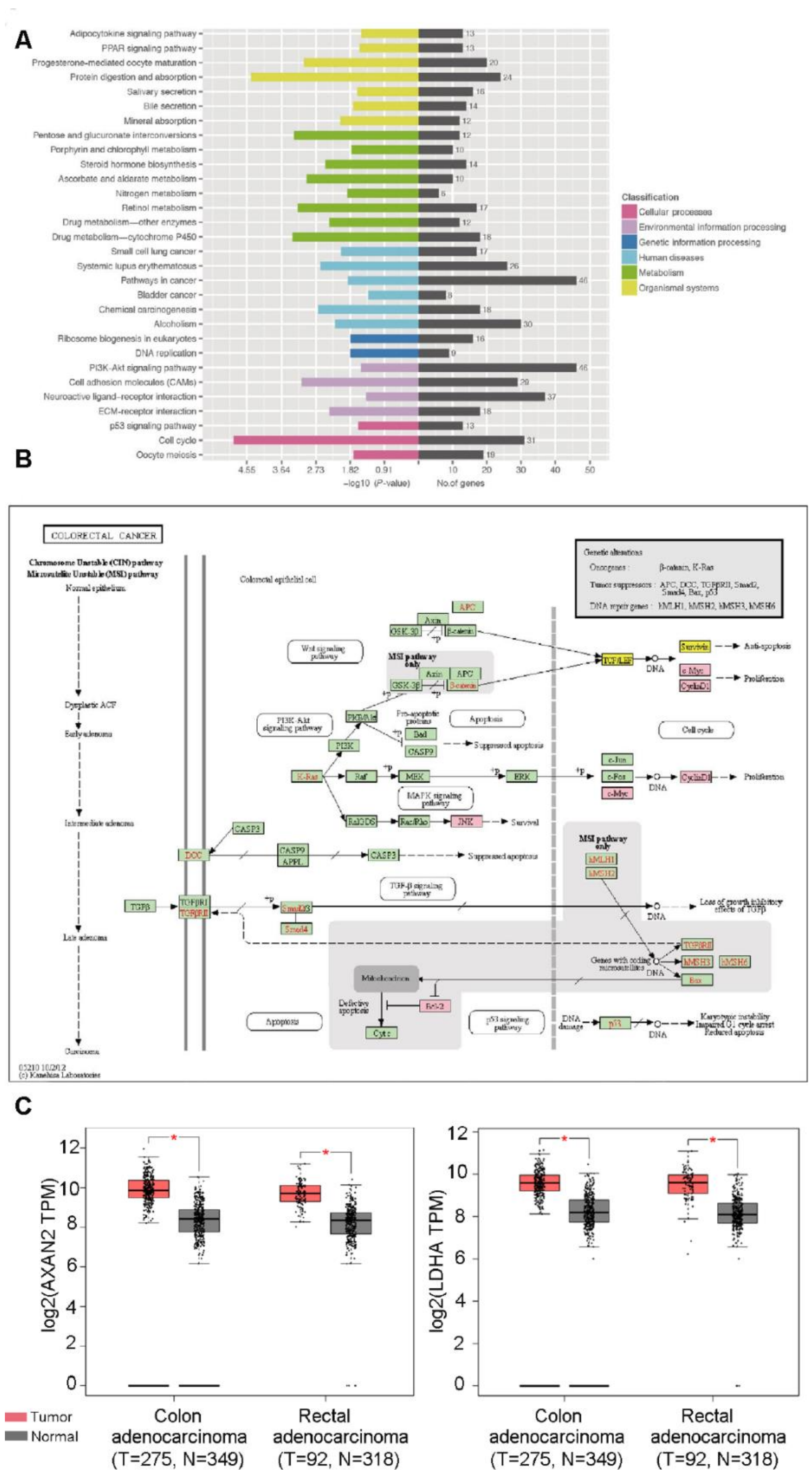

Supplementary Figure 3. (**A**) Enrichment statistics of KEGG pathways in a sample-classification map. (**B**) The CRC signaling pathways. (**C**) The expression of AXAN2/LDHA. Data were collected from the TCGA and GTEx databases using GEPIA (http://gepia.cancer-pku.cn/index.html).

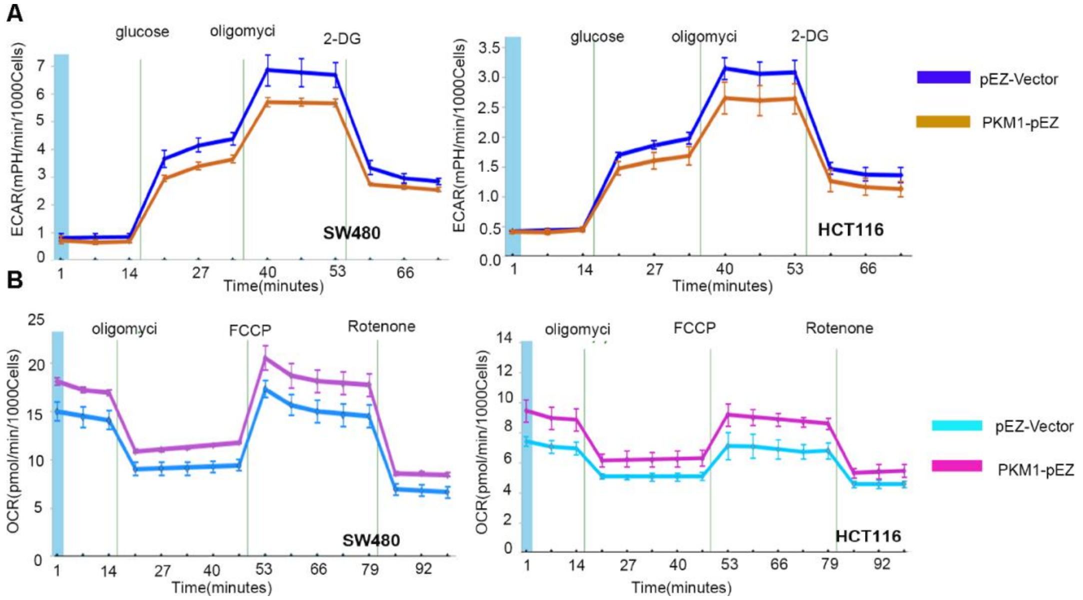

Supplementary Figure 4. (**A**) The ECAR and (**B**) OCR of cells transfected with NC or Si2 was measured. The statistical analysis was performed using the two-tailed Student's t-test. $^{**}P < 0.01$, $^{***}P < 0.001$.
